# Combining shear wave elastography and ultrasound localization microscopy for liver fibrosis staging in a rat model

**DOI:** 10.1186/s41747-026-00841-9

**Published:** 2026-11-06

**Authors:** Yue Zhang, Haoming Lin, Xinli Kong, Huiying Wen, Zhongjun Ma, Mian Chen, Siping Chen, Xin Chen, Zhong Liu

**Affiliations:** National-Regional Key Technology Engineering Laboratory for Medical Ultrasound, Guangdong Key Laboratory of Biomedical Measurements and Ultrasound Imaging, School of Biomedical Engineering, Shenzhen University Medical School, Shenzhen, China

**Keywords:** Elasticity imaging techniques, Fibrosis, Microvessels, Rats (Sprague–Dawley), Shear wave elastography, Ultrasound localization microscopy

## Abstract

**Objective:**

Accurate staging of liver fibrosis (LF) remains a clinical challenge. This study aimed to quantitatively evaluate the diagnostic value of combining shear wave elastography (SWE) and ultrasound localization microscopy (ULM) for LF staging across the full disease spectrum. We sought to integrate liver mechanical properties and microvascular structural parameters to build a multiparametric model for fibrosis staging.

**Materials and methods:**

LF was induced in Sprague–Dawley rats and histologically staged according to the METAVIR system. A total of 62 rats were included in the final analysis. Both SWE and ULM were performed in each animal. Liver viscoelastic moduli were estimated from SWE data using SW-VEI-Net, a physics-informed neural network-based approach. ULM data were post-processed to reconstruct super-resolution images of the hepatic microvasculature, followed by quantitative analysis to extract microvascular structural parameters.

**Results:**

Histology revealed fibrosis stages F0 (*n* = 10), F1 (*n* = 12), F2 (*n* = 19), F3 (*n* = 11), and F4 (*n* = 10). Compared with the single-modality SWE and ULM models, the multiparametric combined model achieved the highest numerical areas under the receiver operating characteristic curve across the four classification tasks: 0.93 for F0 *versus* F1–F4, 0.92 for F0–F1 *versus* F2–F4, 0.90 for F0–F2 *versus* F3–F4, and 0.89 for F0–F3 *versus* F4.

**Conclusion:**

Combining SWE-derived viscoelastic parameters with ULM-derived microvascular features enabled multiparametric characterization of LF in this preclinical rat model. Independent validation in larger animal cohorts and, ultimately, human studies is essential before robust clinical conclusions can be drawn.

**Key Points:**

***Question*** Can combining shear-wave elastography (SWE) and ULM improve the accuracy of LF staging?

***Findings*** By integrating viscoelastic and microvascular features, the SWE-ULM combined model achieved the highest numerical AUCs across all four fibrosis classifications in a rat model.

***Relevance statement*** The SWE and ULM combined model suggests potential value for LF staging by integrating viscoelastic and microvascular biomarkers, providing a basis for further investigation toward future clinical translation.

**Graphical Abstract:**

**Figure:**
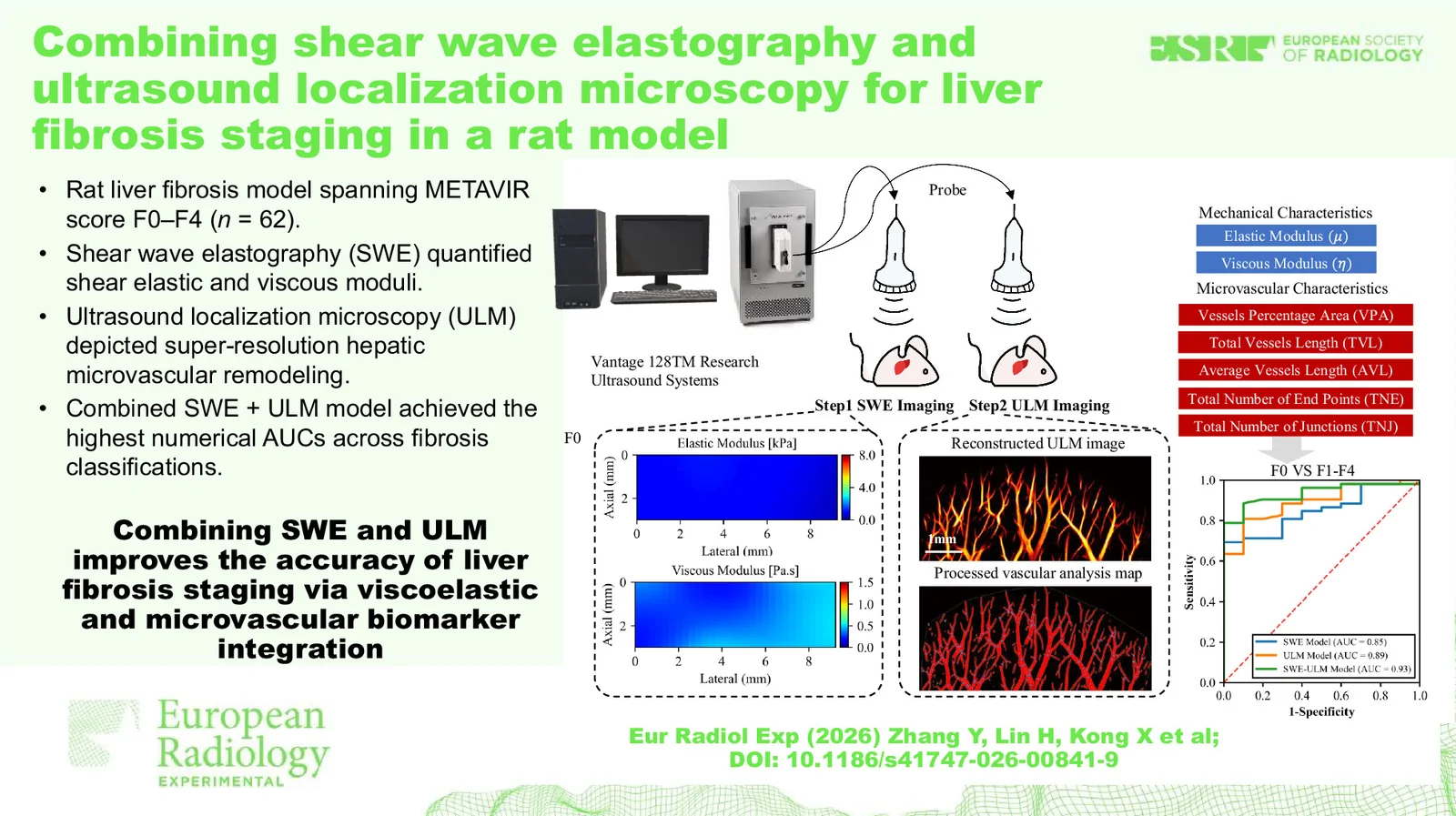

## Introduction

### Background

Chronic liver disease (CLD) represents a growing global health concern, contributing significantly to morbidity and mortality worldwide [1]. A hallmark of CLD progression is the development of liver fibrosis (LF), a wound-healing response characterized by the excessive deposition of extracellular matrix [2]. In the absence of timely intervention, LF may progress to cirrhosis, portal hypertension, and hepatocellular carcinoma [3]. However, it is now widely recognized that LF, and even early cirrhosis, is potentially reversible with timely therapeutic interventions [4]. These observations underscore the need for reliable methods for early and accurate staging of LF. Currently, liver biopsy remains the clinical gold standard for LF staging [5]; however, its invasive nature, potential for sampling error, and associated complications limit its widespread application, particularly for longitudinal monitoring. These limitations have driven the development of noninvasive medical imaging methods, among which ultrasound is particularly attractive because it is widely available, cost-effective, and suitable for repeated examinations.

LF progression is fundamentally linked to changes in the liver’s mechanical properties [6]. The accumulation of fibrotic scar tissue increases liver stiffness [7]. Shear wave elastography (SWE) has become a widely used ultrasound technique to quantify these changes [8]. By measuring the speed of shear-wave propagation through the tissue, SWE provides a quantitative mechanical metric strongly correlated with the degree of LF. The application of SWE in the diagnosis and staging of LF has been extensively investigated in previous studies [9, 10], affirming its clinical utility. The time-of-flight method [8], the most commonly used algorithm for shear wave speed estimation, can only derive the elastic modulus, neglecting the influence of viscosity on liver stiffness. To address this limitation, shear wave dispersion-based methods (*e.g*., SDUV [11] and MUSIC [12]) have been developed to simultaneously obtain both the elastic modulus and viscous modulus. These methods depend on robust estimation of shear wave phase velocity. However, phase velocity estimation, particularly at high frequencies, is highly sensitive to noise, which ultimately leads to unstable results [13]. Recently, physics-informed neural networks (PINNs), specialized architectures integrating physical principles, have been proposed for SWE [14]. In a recent study [15], SW-VEI-Net, a novel PINN-based framework for quantitative viscoelasticity imaging has been developed. This innovative framework integrates the viscoelastic wave equation into a dual-network architecture, enabling the simultaneous reconstruction of both the shear elastic modulus and viscous modulus, and achieving more accurate and stable estimation.

In parallel with mechanical alterations, LF also induces substantial remodeling of the hepatic microvascular architecture [16]. These changes, including vessel tortuosity, rarefaction, and the formation of vascular shunts, are critical features of disease progression [17, 18]. Ultrasound localization microscopy (ULM) is an innovative super-resolution imaging technique that leverages contrast agents by localizing and tracking individual microbubbles within the vasculature, thereby enabling vascular visualization at the micrometer scale [19, 20]. ULM has been applied to the assessment of microvascular architecture in a variety of organs and tumors, including the brain [19, 21], glioblastoma [22], kidney [23], and breast tumors [24]. Research has also employed ULM to visualize microvascular architecture and hemodynamic changes during liver regeneration [25]. These successful applications motivated us to use ULM to characterize subtle microvascular changes across different stages of LF, thereby providing insight into the underlying disease pathophysiology.

Medical imaging technology is exhibiting a clear trend toward multimodality, and the fusion of multimodal information is poised to provide critical complementary insights for disease diagnosis [26]. In the field of CLD diagnosis, previous studies have combined SWE-derived quantitative measures with serological indicators to improve diagnostic performance in patients with chronic hepatitis B [27]. Furthermore, other researchers have also achieved simultaneous assessment of LF, inflammation, and steatosis by extracting multimodal tissue characterization parameters from B-mode images, two-dimensional SWE images, and radiofrequency signals [28]. Collectively, these studies suggest that multimodal evaluation may offer advantages over single-modality assessment for the comprehensive characterization of liver disease. LF is a complex, multi-faceted process involving not only changes in tissue stiffness but also significant remodeling of the microvascular network. To our knowledge, no study has combined quantitative parameters from SWE and ULM for LF staging.

In this study, we aimed to address this research gap by developing and evaluating a novel multiparametric approach. We hypothesized that a combined model integrating quantitative parameters from both imaging techniques would provide a more accurate and robust assessment of LF across the full disease spectrum in a rat model.

## Materials and methods

### Animal model

This preclinical study was designed as a cross-sectional diagnostic study in a CCl4-induced rat model of LF. All experimental procedures involving animals were performed according to the National Institutes of Health Guide for the Care and Use of Laboratory Animals and were approved by the Ethics Committee of Shenzhen University (Ethical approval No. IACUC-202300051). Male Sprague–Dawley rats (180–220 g) were housed in a specific pathogen-free facility of the Guangdong Provincial Medical Experimental Animal Center. LF was induced by subcutaneous injection of 50% CCl4 in olive oil into the dorsal region at a dose of 3 mL/kg body weight and the initial priming dose was doubled (6 mL/kg). The injection was given twice a week, and the dosage was adjusted each time according to the body weight variation. The control group received an equal volume of 0.9% saline. In the CCl4 model group, eight rats were scheduled for ultrasound imaging and histological assessment at each time point (weeks 0, 2, 4, 6, 8, 10, 12, and 14). In the control group, one rat was scheduled for assessment at each time point (weeks 2, 6, 10, and 14). A total of 62 rats with complete ultrasound imaging and histological data were included in the final analysis, and fibrosis stages were assigned according to histopathological METAVIR scoring.

### Data acquisition

The rats were anesthetized with an intraperitoneal injection of pentobarbital sodium (30 mg/kg) and placed on a warming pad set to 37 °C to maintain body temperature. During the ultrasound imaging procedure, the animal was continuously anesthetized using an anesthesia machine delivering isoflurane at a maintenance concentration of 1.5% vaporized in medical air (flowing at 1.0 L/min) via a nose cone.

Ultrasound imaging was performed using a Vantage 128 research ultrasound system (Verasonics Inc., Kirkland, WA, USA). For SWE, an L7-4 linear array transducer (Verasonics Inc., Kirkland, WA, USA) was used. For ULM, an L38-22 linear array transducer (Kolo Medical, San Jose, CA, USA) was used. To minimize motion artifacts, the liver was exposed by laparotomy, and the probe was secured using a probe holder during data acquisition. Imaging was consistently focused on the right liver lobe to standardize data collection.

For SWE, the L7-4 linear array transducer was positioned approximately 2 cm above the right liver lobe. The region of interest (ROI) was first determined using B-mode imaging with a center frequency of 5 MHz. After that, the imaging mode was switched to SWE measurement. Mach cone shear wave patterns were generated by a focused acoustic radiation force push beam (25-element aperture, 60 V excitation voltage) using five sequential push pulses (100-µs pulse duration, 200-µs inter-pulse duration, and 3 mm inter-pulse depth excitation interval). The excitation center of the middle push spot was spatially aligned with the center of the ROI in depth. Shear-wave propagation was tracked using angle-compounded plane-wave imaging with steering angles of -4°, -2°, 0°, +2°, +4°. Coherent compounding yielded an effective frame rate of 2 kHz. For each rat, the raw in-phase/quadrature data were collected during the SWE sequence. Six repeated SWE datasets were acquired from each rat under the same imaging protocol.

For ULM, the high-frequency linear array transducer (L38–22) was utilized. To achieve a consistent scanning plane across all rats, the probe was positioned parallel to the longitudinal axis of the right liver lobe and carefully centered over the main lobe. B-mode imaging was then used to define the final imaging plane while avoiding large vessels and lobe margins. Microbubble (SonoVue®, Bracco Imaging, Italy) suspensions (2.5 × 10^7^ microbubbles/mL in saline) were continuously infused into the tail vein at a constant rate of 30 µL/min. The ULM imaging was performed with a center frequency of 31.25 MHz, employing angle-compounded plane-wave imaging with steering angles of -4°, -2°, 0°, +2°, +4°, yielding an effective frame rate of 1.25 kHz. For each rat, 150 groups of in-phase/quadrature data were collected, with each group containing 1,000 frames, resulting in a total of 150,000 data frames.

### Data processing

**Fig. 1:**
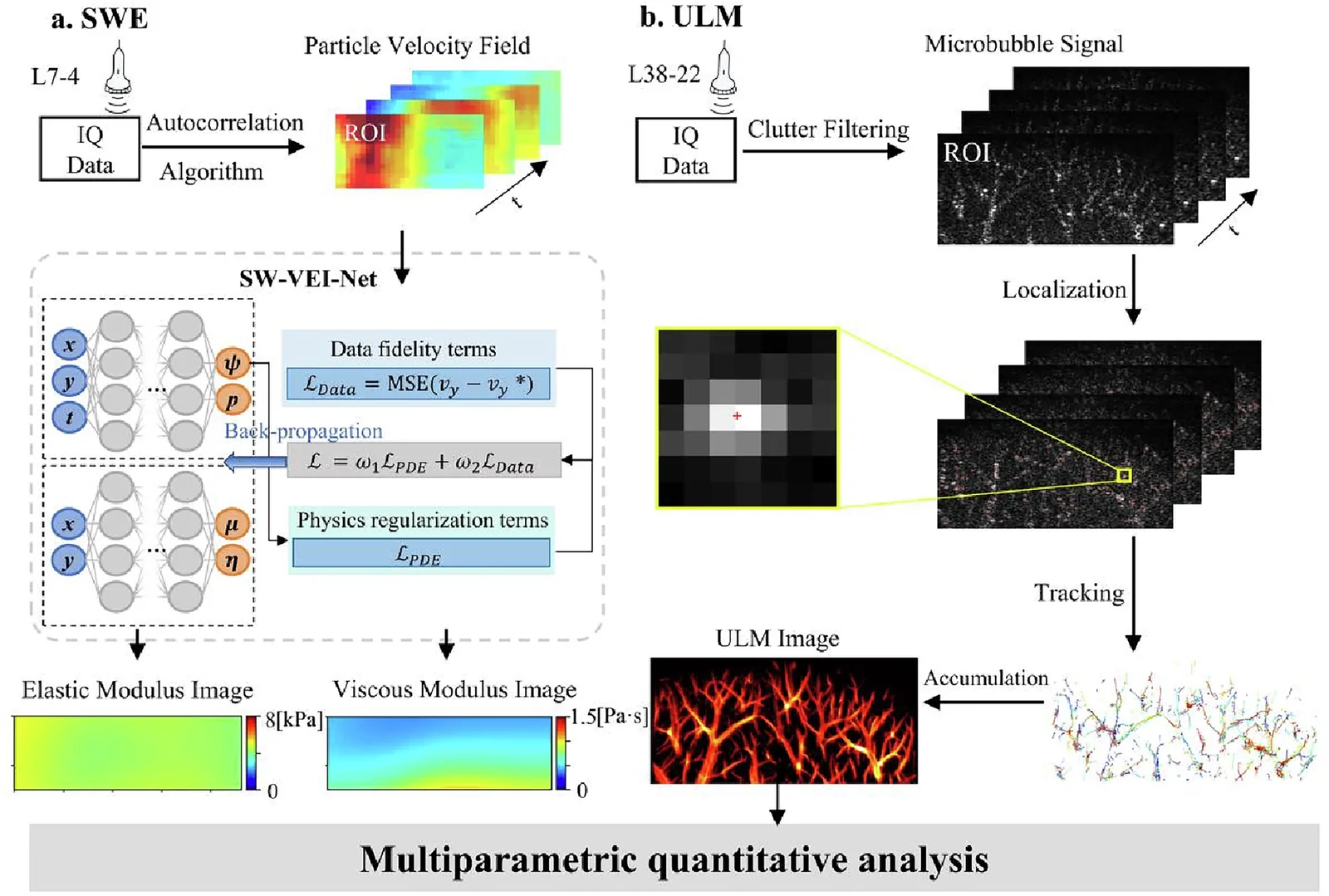
Multiparametric ultrasound imaging workflow. **a** Workflow of SWE. IQ data sequences were processed using one-dimensional autocorrelation to estimate tissue particle velocity fields, which were then input into SW-VEI-Net to reconstruct viscoelastic parameter maps. **b** Workflow of ULM. Microbubble signals were extracted from IQ data by clutter filtering and reconstructed into super-resolution microvascular images through localization, tracking, and accumulation. The combination of viscoelastic and vascular parameters enabled comprehensive multiparametric quantitative analysis. SWE, Shear wave elastography; ULM, Ultrasound localization microscopy; IQ, In-phase/quadrature

Figure 1 illustrates the workflow for SWE and ULM data processing. For SWE data, raw in-phase/quadrature data were processed using a one-dimensional autocorrelation algorithm to estimate shear-wave particle vibration velocity [29], followed by spatial filtering for noise reduction. The filtered velocity fields were input into SW-VEI-Net, a PINN based on the Kelvin-Voigt viscoelastic model, to simultaneously reconstruct the shear elastic modulus (μ) and viscous modulus (η) [15]. For each rat, ROI-averaged μ and η values were calculated from six repeated SWE acquisitions and averaged to obtain one per-animal value for subsequent statistical analysis and modeling. Detailed SW-VEI-Net architecture, loss functions, and reconstruction implementation are provided in Supplementary Methods S1.

For ULM data, raw in-phase/quadrature sequences were processed using a pipeline including motion-corrupted frame rejection, singular value decomposition filtering and temporal filtering for microbubble signal extraction [30], radial symmetry-based microbubble localization [31], Kuhn–Munkres trajectory tracking [32], and trajectory accumulation to reconstruct super-resolution microvascular images. A standardized subcapsular liver ROI was applied consistently across all animals, and microvascular features, including vessel percentage area (VPA), total vessel length (TVL), average vessel length (AVL), total number of junctions (TNJ), and total number of endpoints (TNE), were extracted using AngioTool [33, 34]. Detailed filtering parameters, localization and tracking settings, interpolation procedures, and ROI definitions are provided in Supplementary Methods S2.

### Histological analyses

After ultrasound imaging, the rats were humanely euthanized using carbon dioxide inhalation. Liver tissue samples were then immediately excised from the right liver lobe for histological examination. The samples were rinsed briefly with phosphate-buffered saline to remove excess blood, then fixed in 10% neutral-buffered formalin for a mandatory 24-h period and embedded in paraffin. LF was evaluated using hematoxylin and eosin staining and Sirius red staining according to the METAVIR scoring system: F0, no fibrosis; F1, fibrous portal expansion; F2, few bridges or septa; F3, obvious bridging fibrosis; and F4, cirrhosis [35]. Histopathologic evaluation and METAVIR fibrosis staging were performed once by an experienced pathologist. The pathologist reviewed all slides independently, completely blinded to the animal groups and all ultrasound imaging results.

### Statistical analyses and classification modeling

The distribution of all quantitative metrics was assessed for normality using the Shapiro–Wilk test. Given that not all data followed a normal distribution, continuous variables were presented as medians with interquartile ranges (IQR). Multiple-group comparisons were performed using the Kruskal–Wallis test followed by Dunn’s post hoc test, while comparisons between two groups were conducted using the Mann–Whitney *U*-test. For comparisons of SWE-derived and ULM-derived parameters across various LF groupings, *p*-values were adjusted using the Benjamini–Hochberg false discovery rate procedure. A *p*-value < 0.05 was considered statistically significant. GraphPad Prism (version 8.0, GraphPad Software, USA) was used for statistical analyses and data display.

To comprehensively evaluate the diagnostic performance, several binary classification tasks were defined to mirror critical clinical and biological milestones in hepatology: ≥ F1 (initial matrix deposition/early screening), ≥ F2 (septal extension/threshold for pharmacological intervention), ≥ F3 (bridging fibrosis/intensified monitoring), and F4 (cirrhosis/strict surveillance for hepatocellular carcinoma and portal hypertension).

The diagnostic performance for staging LF was evaluated using machine-learning-based classification models. Three distinct models were developed based on the input features: an SWE model (using elasticity and viscosity), a ULM model (using ULM-derived parameters VPA, TVL, AVL, TNJ, and TNE), and an SWE-ULM combined model integrating all aforementioned SWE and ULM parameters. Given the predefined biological imaging features, no additional automated feature selection was applied. All models were trained and evaluated using stratified five-fold cross-validation with fold-wise feature normalization. The core classifier used a balanced bagging ensemble with a Random Forest base estimator. Classification thresholds were optimized within each training fold using Youden’s index and then applied to the held-out test fold. Diagnostic metrics were calculated from pooled out-of-fold predictions. Area under the receiver operating characteristic curve (AUC) 95% confidence intervals were estimated using bootstrap resampling, and pairwise AUC differences between models were assessed using the DeLong test [36]. The calibration performance of various models was assessed in terms of the Brier score [37]. Detailed hyperparameters, class-imbalance handling, and exploratory internal validation procedures are provided in Supplementary Methods S3.

## Results

### Histological results

**Fig. 2:**
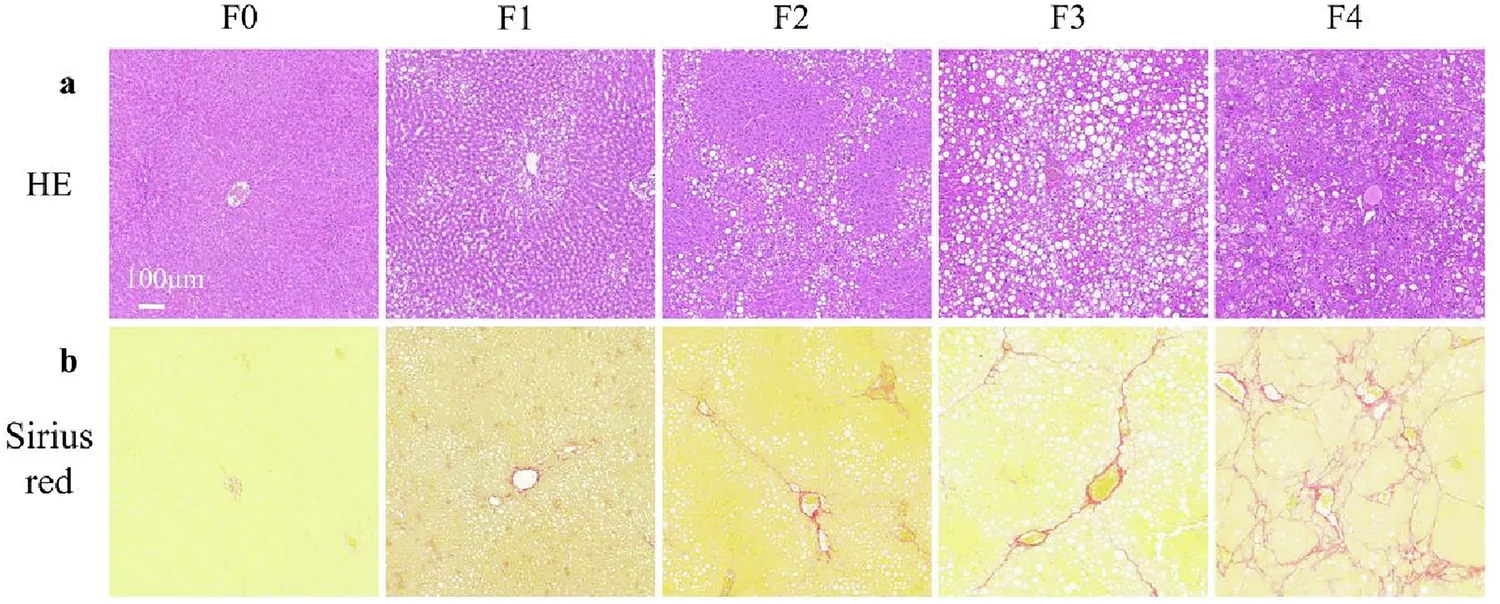
Representative histopathological images across LF stages (F0–F4). **a** H&E staining. **b** Sirius red staining. Scale bars: 100 µm. H&E, Hematoxylin and eosin

Figure 2 presents representative histological alterations in rat liver tissue at different fibrosis stages (F0 to F4) through two staining methods: hematoxylin and eosin (Fig. 2a) and Sirius red (Fig. 2b).

At stage F0, hematoxylin and eosin staining revealed a normal liver architecture with well-preserved hepatocyte lobules. As the fibrosis progressed from F1 to F4, tissue disorganization, hepatocyte degeneration, and fibrotic lesions gradually increased, with a severe disruption of the architecture at F4 (Fig. 2a). Sirius red staining indicated minimal collagen deposition at F0, and the collagen accumulation increased progressively as the fibrosis stage advanced, ultimately forming a dense collagen network at F4 (Fig. 2b).

According to the histopathologic evaluation and METAVIR fibrosis staging, the final histological distribution was as follows: F0 (*n* = 10), F1 (*n* = 12), F2 (*n* = 19), F3 (*n* = 11), and F4 (*n* = 10).

### Quantitative results of SWE

**Fig. 3:**
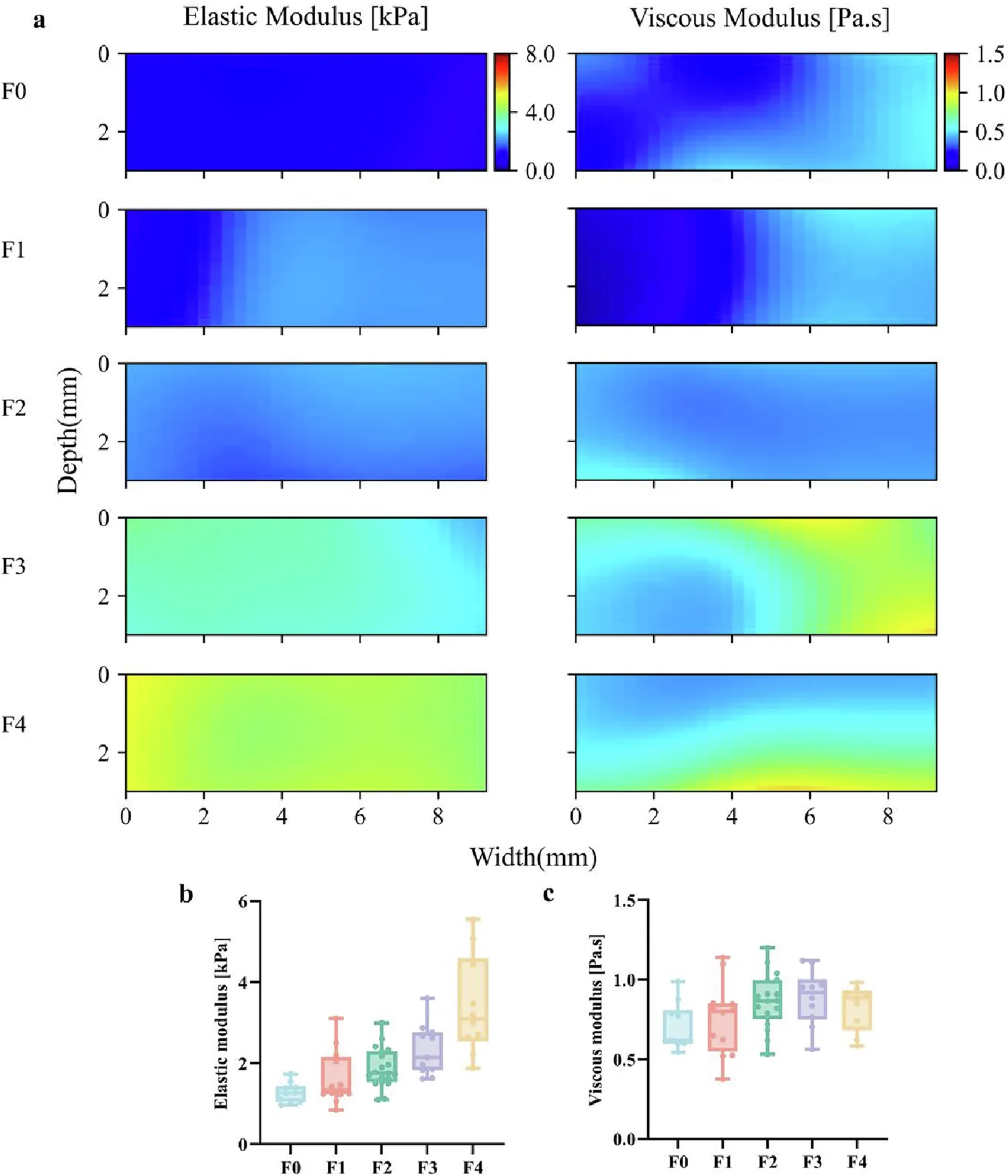
Viscoelastic maps and quantitative comparisons across LF stages (F0–F4). **a** Representative spatial distributions of the shear elastic modulus (μ, kPa; left column) and viscous modulus (η, Pa·s; right column) in rat liver from F0 to F4. **b**, **c** Box plots summarizing the estimated μ in **b** and η in **c** across stages. Box borders represent the 25th and 75th percentiles, midlines indicate the median, and whiskers denote the minimum and maximum values

The SWE-derived viscoelastic parameters, the shear elastic modulus (μ) and the viscous modulus (η), were analyzed across fibrosis stages (F0–F4). Specifically, Fig. 3a shows representative spatial maps of the shear elastic and viscous moduli for each fibrosis stage, providing a visual illustration of the stage-dependent changes in liver viscoelastic properties; corresponding B-mode images are provided as anatomical references for the approximate SWE imaging regions and acoustic radiation force push focal locations (Supplementary Fig. S1). With increasing fibrosis severity, the elastic modulus maps exhibited a gradual elevation in overall magnitude, whereas the viscous modulus maps displayed relatively subtle variations across stages.

Quantitative values are summarized in Fig. 3b, c and Table 1. The shear elastic modulus exhibited a progressive increase corresponding to the severity of LF, indicating a gradual stiffening of the liver tissue (μ [kPa]: F0, 1.27 [IQR, 1.05–1.44]; F1, 1.34 [1.23–2.16]; F2, 1.75 [1.53–2.30]; F3, 2.14 [1.83–2.76]; and F4, 3.09 [2.54–4.59] [*p* < 0.001]). In contrast, the viscous modulus showed limited overall variation across the individual fibrosis stages (η [Pa·s]: F0, 0.62 [0.60–0.81]; F1, 0.80 [0.55–0.85]; F2, 0.87 [0.75–1.00]; F3, 0.92 [0.75–1.00]; and F4, 0.89 [0.68–0.93] [*p* = 0.071]). However, the η values in significant fibrosis stages (F2–F4) were noticeably higher than those in the non-significant stages (F0–F1). While this trend did not reach statistical significance in the overall multi-group comparison, it suggests that the viscous modulus may still hold potential value in monitoring the progression of fibrosis.

**Table 1:** SWE and ULM parameters across LF stages (F0–F4)

	F0	F1	F2	F3	F4	*p*
μ (kPa)	1.27 (1.05–1.44)	1.34 (1.23–2.16)	1.75 (1.53–2.30)	2.14 (1.83–2.76)^*^	3.09 (2.54–4.59)^*†‡^	< 0.001
η (Pa·s)	0.62 (0.60–0.81)	0.80 (0.55–0.85)	0.87 (0.75–1.00)	0.92 (0.75–1.00)	0.89 (0.68–0.93)	0.071
VPA (%)	24.6 (23.1–26.0)	24.7 (22.9–30.8)	30.4 (27.3–31.1)	31.4 (30.1–32.8)	36.6 (34.0–40.3)^*†^	< 0.001
TVL (mm)	73.6 (70.6–87.9)	77.3 (72.2–91.0)	91.3 (83.1–95.9)	86.2 (73.3–106.2)	110.3 (94.6–123.3)^*†^	0.003
AVL (mm)	1.63 (0.75–2.37)	1.12 (0.85–1.42)	0.88 (0.69–1.24)	0.73 (0.50–1.39)	0.86 (0.71–0.99)	0.120
TNJ (pieces)	166 (115–211)	198 (160–211)	229 (210–248)	294 (268–312)^*^	351 (307–406)^*†‡^	< 0.001
TNE (pieces)	276 (220–307)	290 (283–366)	377 (322–428)^*^	402 (360–634)^*^	569 (507–655)^*†^	< 0.001

Unless otherwise indicated, data are medians, with IQRs in parentheses. Statistical differences among groups were determined with the Kruskal–Wallis test. The *p*-value column is for comparison among all groups

*AVL* Average vessel length, *IQRs* Interquartile ranges, *SWE* Shear wave elastography, *TNE* Total number of endpoints, *TNJ* Total number of junctions, *TVL* Total vessel length, *ULM* Ultrasound localization microscopy, *VPA* Vessel percentage area

^*^ *p* < 0.05 *versus* the F0 group

^†^ *p* < 0.05 *versus* the F1 group

^‡^ *p* < 0.05 *versus* the F2 group

### Quantitative results of ULM

**Fig. 4:**
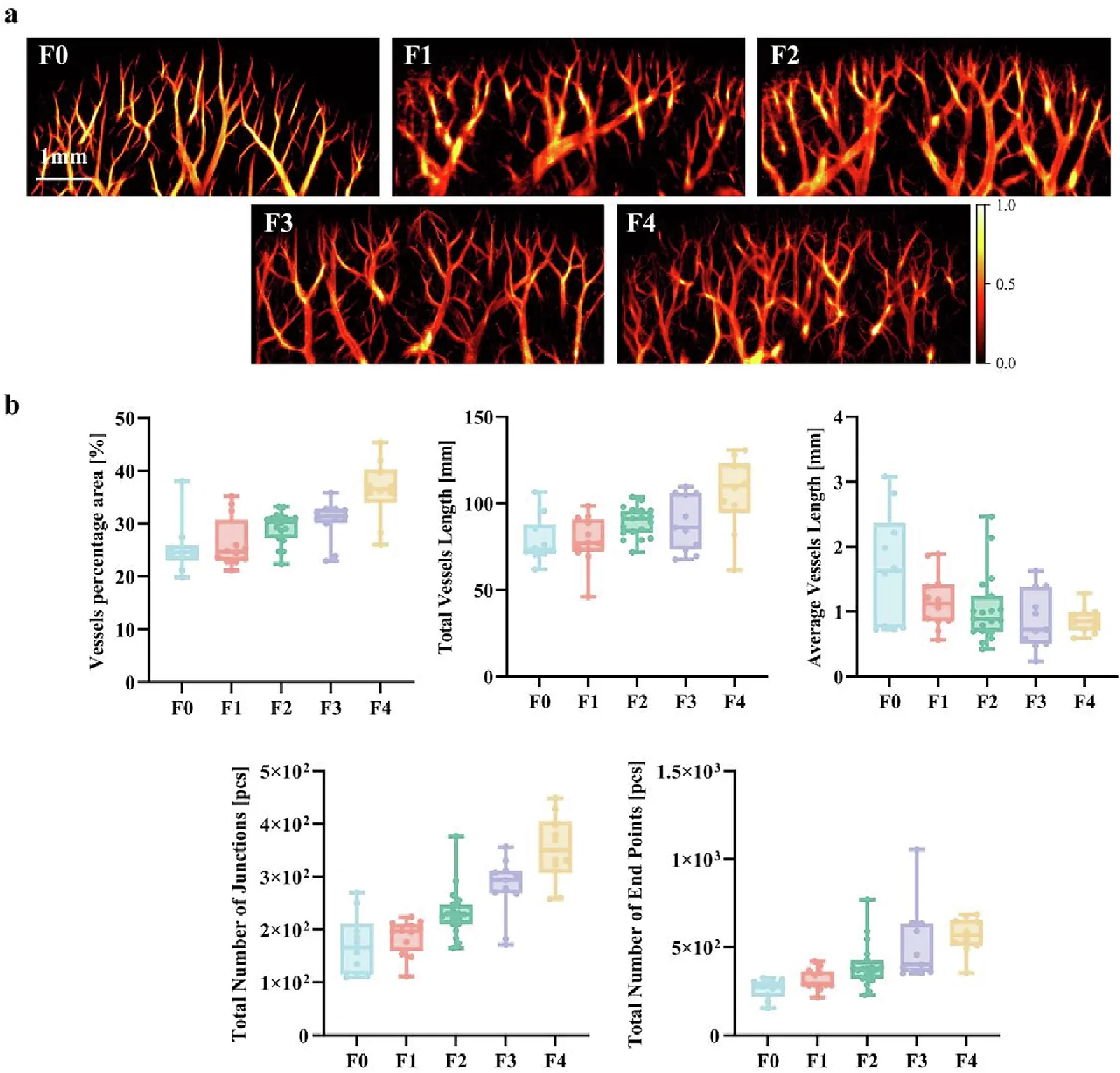
Super-resolution microvascular images and quantitative comparisons across LF stages (F0–F4). **a** Representative super-resolution images of microvasculature in rat liver from F0 to F4. Scale bars: 1 mm. **b** Box plots of five microvascular parameters from F0 to F4. Box borders represent the 25th and 75th percentiles, midlines indicate the median, and whiskers denote the minimum and maximum values

Representative ULM images illustrate the liver microvascular network across fibrosis stages (F0–F4) (Fig. 4a), with corresponding B-mode images provided as anatomical references for the approximate ULM imaging regions (Fig. S2). In the F0 stage, the microvasculature presented a highly organized branching architecture. As fibrosis progressed through F1, F2, F3, and F4, the vascular structure gradually became disorganized, with noticeable alterations in vessel density and branching patterns, especially in the advanced F4 stage.

Quantitative microvascular parameters are summarized in Fig. 4b and Table 1. With the progression of fibrosis, both VPA and TVL exhibited a general upward trend, consistent with increased vascular area and vessel length. (VPA [%]: F0, 24.6 [23.1–26.0]; F1, 24.7 [22.9–30.8]; F2, 30.4 [27.3–31.1]; F3, 31.4 [30.1–32.8]; and F4, 36.6 [34.0–40.3] [*p* < 0.001]; TVL [mm]: F0, 73.6 [70.6–87.9]; F1, 77.3 [72.2–91.0]; F2, 91.3 [83.1–95.9]; F3, 86.2 [73.3–106.2]; and F4, 110.3 [94.6–123.3] [*p* = 0.003]). Conversely, the AVL displayed a downward trend, suggesting increased vessel fragmentation (AVL [mm]: F0, 1.63 [0.75–2.37]; F1, 1.12 [0.85–1.42]; F2, 0.88 [0.69–1.24]; F3, 0.73 [0.50–1.39]; F4, 0.86 [0.71–0.99] [*p* = 0.120]). Concurrently, the complexity of the vascular network intensified, as evidenced by significant increases in both the TNJ and TNE (TNJ [pieces]: F0, 166 [115–211]; F1, 198 [160–211]; F2, 229 [210–248]; F3, 294 [268–312]; and F4, 351 [307–406] [*p* < 0.001]; TNE [pieces]: F0, 276 [220–307]; F1, 290 [283–366]; F2, 377 [322–428]; F3, 402 [360–634]; and F4, 569 [507–655] [*p* < 0.001]).

### Comparative diagnostic performance for LF staging

For further analysis, statistical comparisons were performed across all four clinically relevant LF groupings. All comparisons of SWE and ULM parameters across these groupings are summarized in Table 2.

**Table 2:** Comparison of SWE-derived and ULM-derived parameters across four clinically relevant LF groupings

	F0 *versus* F1–F4			F0–F1 *versus* F2–F4			F0–F2 *versus* F3–F4			F0–F3 *versus* F4		
	F0	F1–F4	*p*	F0–F1	F2–F4	*p*	F0–F2	F3–F4	*p*	F0–F3	F4	*p*
μ (kPa)	1.27 (1.05–1.44)	1.96 (1.55–2.67)	0.002	1.27 (1.10–1.59)	2.16 (1.71–2.75)	0.002	1.53 (1.25–2.00)	2.67 (1.92–3.33)	0.002	1.68 (1.26–2.19)	3.09 (2.54–4.59)	0.002
η (Pa·s)	0.62 (0.60–0.81)	0.85 (0.71–0.95)	0.030	0.71 (0.60–0.85)	0.89 (0.75–0.96)	0.008	0.81 (0.62–0.91)	0.89 (0.74–0.95)	0.169	0.82 (0.62–0.95)	0.89 (0.68–0.93)	0.670
VPA (%)	24.6 (23.1–26.0)	30.9 (25.5–33.1)	0.013	24.7 (23.2–26.4)	31.1 (28.8–33.2)	0.002	26.8 (24.2–31.0)	32.8 (30.5–36.6)	0.002	28.9 (24.4–31.5)	36.6 (34.0–40.3)	0.002
TVL (mm)	73.6 (70.6–87.9)	90.2 (77.8–100.7)	0.034	75.5 (71.5–89.5)	92.4 (82.1–104.6)	0.002	83.8 (74.2–93.1)	101.4 (79.0–110.8)	0.014	84.8 (74.0–95.1)	110.3 (94.62–123.3)	0.002
AVL (mm)	1.63 (0.75–2.37)	0.89 (0.70–1.24)	0.044	1.20 (0.77–1.87)	0.87 (0.68–1.06)	0.019	1.01 (0.74–1.55)	0.84 (0.63–1.04)	0.049	0.98 (0.71–1.43)	0.86 (0.71–0.99)	0.304
TNJ (pieces)	166 (115–211)	234 (200–307)	0.002	191 (146–209)	263 (222–329)	0.002	208 (175–231)	312 (269–363)	0.002	214 (179–263)	351 (307–406)	0.002
TNE (pieces)	276 (220–307)	380 (345–540)	0.002	286 (265–330)	395 (365–592)	0.002	322 (280–380)	525 (375–642)	0.002	354 (285–394)	569 (507–655)	0.002

Unless otherwise indicated, data are medians, with IQRs in parentheses. *p*-values were obtained using the Mann–Whitney *U*-test and adjusted using the Benjamini–Hochberg false discovery rate procedure across all univariable comparisons reported in this table. For unadjusted *p* < 0.001, 0.001 was used as a conservative input for adjustment

*AVL* Average vessel length, *IQRs* interquartile ranges, *SWE* Shear wave elastography, *ULM* Ultrasound localization microscopy, *VPA* Vessel percentage area, *TNE* Total number of endpoints *TNJ* Total number of junctions, *TVL* Total vessel length

**Fig. 5:**
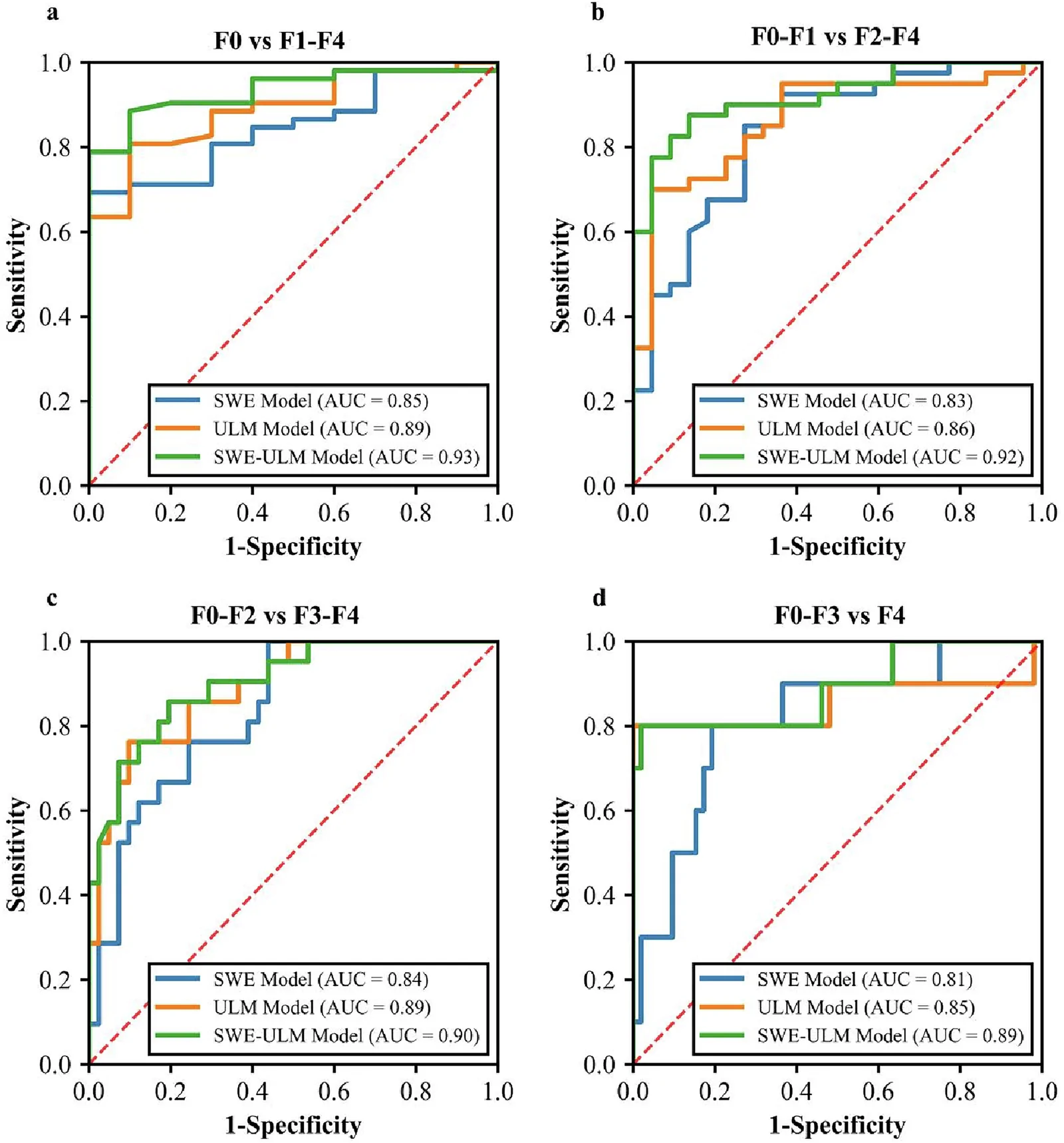
ROC curves of SWE, ULM, and combined SWE-ULM models. The blue line represents the SWE model, the orange line depicts the ULM model, and the green line corresponds to the combined model. **a** Nonfibrotic stage (F0) *versus* fibrotic stage (F1–F4). **b** Nonsignificant fibrosis (F0–F1) vs significant fibrosis (F2–F4). **c** Non-advanced fibrosis (F0–F2) vs advanced fibrosis (F3–F4). **d** Noncirrhotic stage (F0–F3) *versus* cirrhosis (F4). ROC, Receiver operating characteristic; SWE, Shear wave elastography; ULM, Ultrasound localization microscopy

The receiver operating characteristic curves of the SWE, ULM, and SWE-ULM combined models are shown in Fig. 5, and the corresponding diagnostic metrics are summarized in Table 3. The SWE-ULM combined model consistently yielded the highest numerical AUCs across all classifications, although statistically significant differences were observed only in selected pairwise comparisons. Specifically, for F0 *vs* F1–F4 classification, the combined model achieved an AUC of 0.93 ([95% confidence interval 0.86, 0.99]), compared with SWE (0.85 [0.74, 0.94], *p* = 0.041) and ULM (0.89 [0.78, 0.97], *p* = 0.128). For F0–F1 *vs* F2–F4 classification, the AUC reached 0.92 ([0.85, 0.98]), compared with SWE (0.83 [0.72, 0.93], *p* = 0.174) and ULM (0.86 [0.77, 0.95], *p* = 0.048). For F0–F2 *vs* F3–F4 classification, the AUC was 0.90 ([0.80, 0.97]), compared with SWE (0.84 [0.72, 0.92], *p* = 0.103) and ULM (0.89 [0.80, 0.96], *p* = 0.701). For F0–F3 *vs* F4 classification, the AUC was 0.89 ([0.72, 1.00]), compared with SWE (0.81 [0.64, 0.94], *p* = 0.087) and ULM (0.85 [0.63, 1.00], *p* = 0.723). Additional internal validation using repeated stratified five-fold cross-validation showed stable model performance across 30 repetitions, with the SWE-ULM combined model generally showing higher mean AUCs and lower Brier scores (Table S1). Permutation feature-importance analysis further showed that both SWE-derived μ and ULM-derived vascular features, particularly TNE and TNJ, contributed to the combined model across tasks (Table S2). Overall, these results suggest that integrating SWE-derived and ULM-derived features may provide complementary value for LF staging.

**Table 3:** Diagnostic performance of the SWE, ULM, and combined SWE-ULM models for four binary classification tasks

Classification tasks	Model	AUC (95% CI)	Sensitivity	Specificity	Accuracy
F0 vs F1–F4	SWE	0.85 (0.74–0.94)	81%	60%	77%
	ULM	0.89 (0.78–0.97)	87%	70%	84%
	SWE-ULM	0.93 (0.86–0.99)	96%	70%	92%
F0–F1 vs F2–F4	SWE	0.83 (0.72–0.93)	75%	68%	72%
	ULM	0.86 (0.77–0.95)	80%	73%	77%
	SWE-ULM	0.92 (0.85–0.98)	82%	91%	85%
F0–F2 vs F3–F4	SWE	0.84 (0.72–0.92)	57%	90%	79%
	ULM	0.89 (0.80–0.96)	71%	88%	82%
	SWE-ULM	0.90 (0.80–0.97)	71%	90%	84%
F0–F3 vs F4	SWE	0.81 (0.64–0.94)	50%	87%	81%
	ULM	0.85 (0.62–1.00)	80%	98%	95%
	SWE-ULM	0.89 (0.72–1.00)	80%	100%	97%

*AUC* Area under the receiver operating characteristic curve, *SWE* Shear wave elastography, *ULM* Ultrasound localization microscopy

## Discussion

Accurate evaluation of LF remains a key challenge in the clinical management of CLD, as timely and precise assessment is important for guiding treatment decisions and monitoring disease progression. This study developed and evaluated a multiparametric ultrasound strategy integrating SWE and ULM for LF staging in a rat model. The main finding was that the combined model achieved the highest numerical AUCs across all four clinically relevant binary classification tasks, indicating that viscoelastic and microvascular features may provide complementary information for fibrosis staging.

The SWE analysis showed a progressive increase in shear elastic modulus (μ) with advancing LF severity (Fig. 3a), which aligns with a large body of literature establishing liver stiffness as a primary biomarker for fibrosis progression [9, 38]. This monotonic increase reflects the pathological accumulation of extracellular matrix proteins and subsequent tissue stiffening. In contrast, the viscous modulus (η) exhibited a more complex and nonmonotonic pattern (Fig. 3b). Although η differed significantly in two binary classification tasks, it did not reach significant difference in distinguishing non-advanced (F0–F2) from advanced fibrosis (F3–F4) groups (*p* = 0.157). These findings suggest that viscosity may reflect fibrosis-related tissue remodeling [39–41], but its diagnostic performance may be less consistent than that of elasticity.

Meanwhile, ULM provided valuable insights into the microvascular remodeling accompanying LF. Previous investigations have characterized hepatic microvascular alterations during fibrosis progression utilizing imaging modalities. While modalities such as microcomputed tomography [42, 43] and photoacoustic imaging [34, 44] have been used to assess hepatic vasculature, they are limited by *ex vivo* requirements (microcomputed tomography) or constraints in penetration depth and functional specificity (photoacoustic imaging). The use of ULM for structural topological imaging of hepatic microvasculature in fibrosis staging remains largely unexplored. In this study, quantitative ULM analysis revealed distinct trends in microvascular parameters: VPA, TVL, TNJ and TNE all showed a general increasing trend with advancing fibrosis. Conversely, AVL exhibited a decreasing trend (Fig. 4b). These findings align with the pathological understanding of LF [45, 46].

The increases in VPA and TVL, together with the decrease in AVL, are consistent with fibrosis-associated vascular expansion and morphological remodeling, which may occur in the context of chronic inflammation, hypoxia, and increased metabolic demands within fibrotic tissue [47, 48]. Furthermore, the increases in TNJ and TNE suggest greater vascular complexity, disorganization, and branching heterogeneity within the microvascular network. Such altered angioarchitecture has been widely described during progressive fibrosis and may be associated with remodeling of intrahepatic vascular pathways [45, 49]. Accordingly, ULM-derived vascular features may complement SWE-based stiffness measurements by capturing morphological manifestations of fibrosis-associated microvascular remodeling.

The multiparametric combined model achieved the highest numerical AUCs across all four fibrosis classification tasks, suggesting that biomechanical and microvascular information may provide complementary value. Notably, ULM achieved higher numerical AUCs than SWE across all tasks, suggesting that microvascular alterations may provide discriminatory information for fibrosis staging. Meanwhile, the consistently high performance of the combined model and permutation feature-importance results, which identified both SWE-derived μ and ULM-derived TNE and TNJ as influential contributors, support the complementary role of SWE and ULM in fibrosis classification.

Despite these promising results, several limitations warrant consideration. First, this study was conducted in a CCl4-induced rat model, which may not fully represent the heterogeneous etiologies and fibrosis patterns of human CLDs. Additionally, our control group received normal saline rather than the vehicle (olive oil) used for CCl4 administration. Although our primary objective was to correlate ultrasound imaging metrics with histopathological fibrosis staging, the lack of a vehicle-only control limits our ability to strictly rule out potential minor effects of olive oil. Inflammatory activity and steatosis were also not systematically graded, although both may coexist with fibrosis and affect SWE and ULM measurements. Therefore, these potential confounding effects could not be separated from fibrosis-related changes. Second, the machine-learning analysis was performed on a relatively limited dataset with multiple quantitative imaging variables, which may increase the risk of overfitting and optimistic performance estimation. Although stratified cross-validation, fold-wise preprocessing, training-fold threshold optimization, bootstrap confidence intervals, repeated cross-validation, and Brier score assessment were used to reduce this risk, these analyses should be interpreted as exploratory internal validation only. The limited sample size, particularly in the advanced fibrosis and cirrhosis subgroups, may also have reduced the statistical power to detect modest AUC differences between models in DeLong testing. This limitation is partly attributable to the technical complexity of ULM, which remains an emerging ultrasound imaging technique and requires controlled acquisition conditions, as well as time-consuming reconstruction. These requirements make it challenging to obtain ULM datasets covering the full spectrum of LF in rat models. Larger independent cohorts and external validation are needed to confirm the robustness and generalizability of the proposed classification framework. Third, imaging was performed under controlled conditions with laparotomy and probe stabilization to minimize motion artifacts during ULM acquisition. Therefore, this study must be viewed as a preclinical proof-of-concept study. Independent validation in larger animal cohorts and, ultimately, human studies is essential before robust clinical conclusions can be drawn. Future translation will depend on motion-robust transabdominal ULM acquisition and validation in clinically relevant settings, to improve fibrosis characterization and longitudinal monitoring. Fourth, the reliance on two-dimensional ULM restricted the analysis to a single imaging plane. Although efforts were made to maintain a reproducible scanning plane across animals, minor anatomical variations or angular discrepancies may have introduced sampling bias. Therefore, the future adoption of three-dimensional-ULM [50, 51] may help provide a more comprehensive vascular characterization. Finally, fibrosis staging was performed once by a single experienced pathologist, without formal intraobserver or interobserver agreement assessment, which may introduce a potential subjective bias in the ground truth labels. The selected ULM vascular parameters were also not exhaustive, and additional morphological features, such as vascular tortuosity metrics [17, 52], warrant further investigation. Moreover, while the ULM morphological findings are consistent with fibrosis-associated microvascular remodeling, we did not perform specific vascular histological validations (*e.g*., CD31 endothelial staining). Therefore, these biological interpretations remain observational and require further validation.

## Conclusion

In summary, this study provides preclinical evidence that a multiparametric ultrasound strategy integrating SWE and ULM may improve LF staging by capturing both viscoelastic and microvascular alterations. Independent validation in larger animal cohorts and, ultimately, human studies is essential before robust clinical conclusions can be drawn.

## Supplementary information

**Additional file 1: Table S1.** Repeated stratified five-fold cross-validation results for the SWE, ULM, and SWE-ULM models across the four binary classification tasks. **Table S2.** Permutation feature importance of the combined SWE-ULM model across the four binary classification tasks. **Figure S1.** Representative B-mode images corresponding to SWE measurements across fibrosis stages. Representative B-mode images of rat livers at fibrosis stages F0-F4 acquired during the SWE imaging workflow. Red boxes indicate representative ROI used for SWE viscoelastic parameter estimation. Red crosses indicate the focal locations of the acoustic radiation force push pulses. **Figure S2.** Representative B-mode images corresponding to ULM acquisitions across fibrosis stages. Representative B-mode images of rat livers at fibrosis stages F0-F4 acquired during the ULM imaging workflow. The B-mode images indicate representative regions corresponding to the predefined ULM quantification area.

## Data Availability

The data supporting the findings of this study are available from the corresponding author upon reasonable request.

## Abbreviations

AUC: Area under the receiver operating characteristic curve
AVL: Average vessel length
CLD: Chronic liver disease
IQR: Interquartile range
LF: Liver fibrosis
PINN: Physics-informed neural network
ROI: Region of interest
SWE: Shear wave elastography
TNE: Total number of end points
TNJ: Total number of junctions
TVL: Total vessel length
ULM: Ultrasound localization microscopy
VPA: Vessel percentage area
