# Supplementary material for: Combining shear wave elastography and ultrasound localization microscopy for liver fibrosis staging in a rat model
Source: Eur Radiol Exp. 2026 Nov 6;10:175. doi: 10.1186/s41747-026-00841-9 (PMC13743689; doi:10.1186/s41747-026-00841-9)

Combining shear wave elastography and ultrasound localization microscopy for liver fibrosis staging in a rat model

ELECTRONIC SUPPLEMENTARY MATERIAL

Supplementary Methods S1. SWE data processing and SW-VEI-Net reconstruction

Viscoelastic parameters, including the shear elastic modulus (μ) and viscous modulus (η), were reconstructed using the SW-VEI-Net model [15]. The raw in-phase/quadrature (IQ) data were first processed to estimate the particle vibration velocity of the shear wave using a one-dimensional autocorrelation algorithm [28]. To mitigate speckle noise artifacts in the vibration velocity maps, the estimated particle velocity field was subjected to an 8×8 median filtering operation. The resulting filtered particle vibration velocity served as the primary input for the SW-VEI-Net model.

SW-VEI-Net integrates the governing partial differential equations (PDEs) of shear wave propagation in a Kelvin-Voigt (KV) viscoelastic model and employs a dual-network architecture to simultaneously infer spatially dependent and spatiotemporally dependent parameters. The network was trained by minimizing a composite dual-loss function (L), which explicitly balances two components: data fidelity loss ($\mathrm{L}_{\mathrm{Data}}$) and physics regularization loss (L_{PDE}). The data fidelity loss ensures that the network predictions match the measured data and is defined as the mean squared error (MSE) between the measured velocity field (v_y^*) and the network-predicted velocity field (v_y):

$$\mathrm{L}_{\mathrm{Data}} = MSE(v_y - v_y^*), \tag{S1}$$

The physics regularization loss was defined as $\mathrm{L}_{\mathrm{PDE}} = \mathrm{L}_{\mathrm{PDE1}} + \mathrm{L}_{\mathrm{PDE2}}$, where:

$$\begin{aligned}L_{\mathrm{PDE1}} = \mathrm{MSE}(&\mu(\frac{\partial^2 v_x}{\partial x^2} + \frac{\partial^2 v_x}{\partial y^2}) + \frac{\partial \mu}{\partial y}(\frac{\partial v_x}{\partial y} + \frac{\partial v_y}{\partial x}) \\ &+\eta(\frac{\partial^3 v_x}{\partial x^2 \partial t} + \frac{\partial^3 v_x}{\partial y^2 \partial t}) + 2\frac{\partial \eta}{\partial x}\frac{\partial^2 v_x}{\partial x \partial t} + 2\frac{\partial \mu}{\partial x}\frac{\partial v_x}{\partial x} \\ &+\frac{\partial \eta}{\partial y}(\frac{\partial^2 v_x}{\partial y \partial t} + \frac{\partial^2 v_y}{\partial x \partial t}) - \frac{\partial p}{\partial x} - \rho\frac{\partial^2 v_x}{\partial t^2}),\end{aligned} \tag{S2}$$

$$\begin{aligned}L_{\mathrm{PDE2}} = \mathrm{MSE}(&\mu(\frac{\partial^2 v_y}{\partial x^2} + \frac{\partial^2 v_y}{\partial y^2}) + \frac{\partial \mu}{\partial x}(\frac{\partial v_x}{\partial y} + \frac{\partial v_y}{\partial x}) \\ &+\eta(\frac{\partial^3 v_y}{\partial x^2 \partial t} + \frac{\partial^3 v_y}{\partial y^2 \partial t}) + 2\frac{\partial \eta}{\partial y}\frac{\partial^2 v_y}{\partial y \partial t} + 2\frac{\partial \mu}{\partial y}\frac{\partial v_y}{\partial y} \\ &+\frac{\partial \eta}{\partial x}(\frac{\partial^2 v_x}{\partial y \partial t} + \frac{\partial^2 v_y}{\partial x \partial t}) - \frac{\partial p}{\partial y} - \rho\frac{\partial^2 v_y}{\partial t^2}).\end{aligned} \tag{S3}$$

The total loss is defined as:

$$L = \omega_1 L_{\mathrm{PDE}} + \omega_2 L_{\mathrm{Data}}, \tag{S4}$$

where ω_1 and ω_2 are hyperparameters used to balance the data fidelity and physics consistency terms. Here, the loss weights ω_1 and ω_2 were fixed as 1 and 1, respectively. After the model training and reconstruction, maps of the shear elastic modulus μ and viscous modulus η were obtained over the defined ROI in the liver. For each rat, ROI-averaged μ and η values were calculated from each of the six repeated SWE acquisitions and then averaged to obtain a single per-animal μ and η value for subsequent statistical analysis and modeling. The detailed architecture and implementation of SW-VEI-Net can be found in previous work [15].

Supplementary Methods S2. ULM post-processing and microvascular quantification

For ULM imaging, the acquired IQ data underwent a systematic post-processing pipeline to achieve super-resolution microvascular imaging. First, the IQ data were divided into groups of 1000 frames each. To reduce motion artifacts, frames with a correlation coefficient lower than 0.8 relative to a static reference frame were discarded.

After motion-corrupted frame rejection, singular value decomposition (SVD) filtering and temporal filtering were used to separate microbubble signals from tissue and noise signals [29]. The SVD threshold range was set to 20-800. A Butterworth

bandpass filter with cutoff frequencies of 10-250 Hz was then applied along the slow-time dimension.

Microbubble localization was performed using the radial symmetry (RS) algorithm to estimate microbubble centroids [30]. The point spread function (PSF) of microbubbles was determined by manually selecting a single microbubble within the imaging plane and analyzing its two-dimensional intensity distribution, yielding a 7×7 pixel PSF size for microbubble localization. The Kuhn-Munkres tracking strategy [31] was then employed to link microbubbles in adjacent frames and generate flow trajectories. The minimum trajectory length was set to 15 frames.

Finally, all microbubble flow trajectories were subjected to 10-fold bilinear interpolation and accumulated to reconstruct super-resolution liver vascular images. For ULM quantification, a fixed ROI protocol was applied consistently across all animals. The ROI size was 6.4 mm×3 mm, and the height included the 3-mm subcapsular liver region below the liver capsule. The same ROI definition was used for all animals to ensure consistency in microvascular quantification.

Supplementary Methods S3. Machine-learning implementation and exploratory internal validation

Given the predefined biological imaging features, no additional automated feature selection was applied. All models were trained and evaluated using stratified five-fold cross-validation with a fixed random state. Feature normalization parameters were estimated from the training fold and then applied to the corresponding held-out test fold to avoid information leakage.

The core classifier was built using a balanced bagging ensemble with a Random Forest base estimator. To address class imbalance across fibrosis groupings, random under-sampling of the majority class was dynamically applied during the bootstrapping process of each bagging iteration. Hyperparameters were fixed a priori, including 50 trees for the Random Forest base estimator, a maximum tree depth of 10, and 20 bagging estimators.

Within each training fold, the optimal classification threshold was determined by

maximizing Youden’s index and was then applied to the held-out test fold. Diagnostic performance metrics, including the area under the receiver operating characteristic curve (AUC), sensitivity, specificity, and accuracy were calculated from pooled out-of-fold predictions aggregated across all five test folds. Ninety-five percent confidence intervals for AUC were estimated using bootstrap resampling with 2000 iterations. Pairwise ROC/AUC differences between models were formally assessed using DeLong’s test based on pooled out-of-fold predicted probabilities.

Additional exploratory internal validation was performed using repeated stratified five-fold cross-validation over 30 repetitions. Calibration was assessed using calibration curves and Brier scores. Permutation feature-importance analysis was performed to estimate the relative contribution of each SWE- and ULM-derived feature to the combined model across the four classification tasks.

SUPPLEMENTARY TABLES AND FIGURES

Table S1. Repeated stratified five-fold cross-validation results for the SWE, ULM, and SWE-ULM models across the four binary classification tasks.

Classification Tasks	Model	Repeated CV AUC	Repeated CV Brier Score
**F0 vs F1-F4**	SWE	0.83 ± 0.01	0.180 ± 0.006
	ULM	0.86 ± 0.02	0.137 ± 0.006
	SWE-ULM	0.92 ± 0.01	0.125 ± 0.004
**F0-F1 vs F2-F4**	SWE	0.82 ± 0.02	0.162 ± 0.011
	ULM	0.87 ± 0.01	0.132 ± 0.008
	SWE-ULM	0.94 ± 0.01	0.106 ± 0.006
**F0-F2 vs F3-F4**	SWE	0.82 ± 0.03	0.170 ± 0.013
	ULM	0.91 ± 0.02	0.116 ± 0.010
	SWE-ULM	0.91 ± 0.02	0.115 ± 0.007
**F0-F3 vs F4**	SWE	0.82 ± 0.03	0.148 ± 0.007
	ULM	0.90 ± 0.02	0.095 ± 0.006
	SWE-ULM	0.92 ± 0.01	0.091 ±0.005

Note.— Data are presented as mean ± standard deviation across 30 repeated stratified five-fold cross-validation runs and are provided as supplementary internal validation analyses to assess model stability across different random data partitions. Lower Brier scores indicate lower probability prediction error. Overall, the SWE-ULM model showed the highest mean AUC across tasks and generally the lowest mean Brier scores.

Table S2. Permutation feature importance of the combined SWE-ULM model across the four binary classification tasks.

Classification Tasks	Features	Mean importance
**F0 vs F1-F4**	TNE (pcs)	0.058 ± 0.095
	μ (kPa)	0.045 ± 0.056
	AVL (mm)	0.019 ± 0.037
	η (Pa.s)	0.008 ± 0.032
	VPA (%)	0.006 ± 0.049
	TNJ (pcs)	0.005 ± 0.058
	TVL (mm)	-0.001 ± 0.050

**F0-F1 vs F2-F4**	μ (kPa)	0.090 ± 0.078
	TNE (pcs)	0.084 ± 0.065
	TNJ (pcs)	0.025 ± 0.026
	VPA (%)	0.019 ± 0.028
	η (Pa.s)	0.011 ± 0.027
	AVL (mm)	0.003 ± 0.012
	TVL (mm)	-0.008 ± 0.038
**F0-F2 vs F3-F4**	TNJ (pcs)	0.122 ± 0.047
	μ (kPa)	0.030 ± 0.056
	TVL (mm)	0.016 ± 0.023
	TNE (pcs)	0.016 ± 0.026
	VPA (%)	-0.005 ± 0.025
	η (Pa.s)	-0.010 ± 0.015
	AVL (mm)	-0.015 ± 0.022
**F0-F3 vs F4**	μ (kPa)	0.064 ± 0.130
	TNE (pcs)	0.051 ± 0.114
	TNJ (pcs)	0.030 ± 0.046
	TVL (mm)	0.019 ± 0.042
	VPA (%)	0.008 ± 0.008
	AVL (mm)	0.004 ± 0.008
	η (Pa.s)	0.000 ± 0.000

Note.— Permutation feature importance was calculated within the cross-validation framework using the combined SWE-ULM model for each binary classification task. Importance values represent the mean decrease in AUC after permutation of each feature on the held-out test folds. Higher mean importance values indicate greater contribution to classification performance. These analyses are provided as supplementary model interpretability results to illustrate the relative contribution of SWE-derived and ULM-derived variables. SD, standard deviation.

Figure S1. Representative B-mode images corresponding to SWE measurements across fibrosis stages.

Representative B-mode images of rat livers at fibrosis stages F0-F4 acquired during the SWE imaging workflow. Red boxes indicate representative ROI used for SWE viscoelastic parameter estimation. Red crosses indicate the focal locations of the acoustic radiation force push pulses.

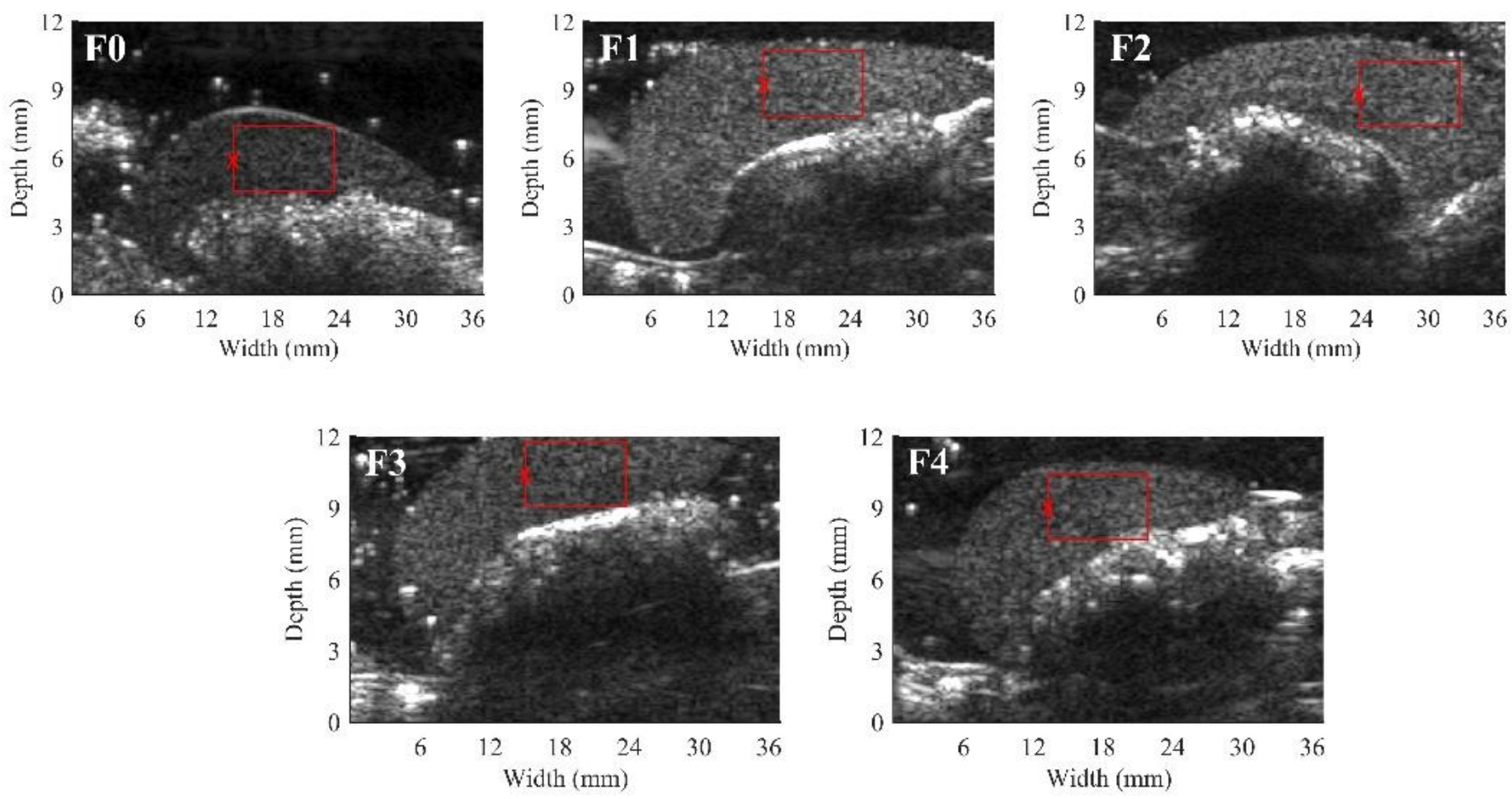

Figure S2. Representative B-mode images corresponding to ULM acquisitions across fibrosis stages.

Representative B-mode images of rat livers at fibrosis stages F0-F4 acquired during the ULM imaging workflow. The B-mode images indicate representative regions corresponding to the predefined ULM quantification area.

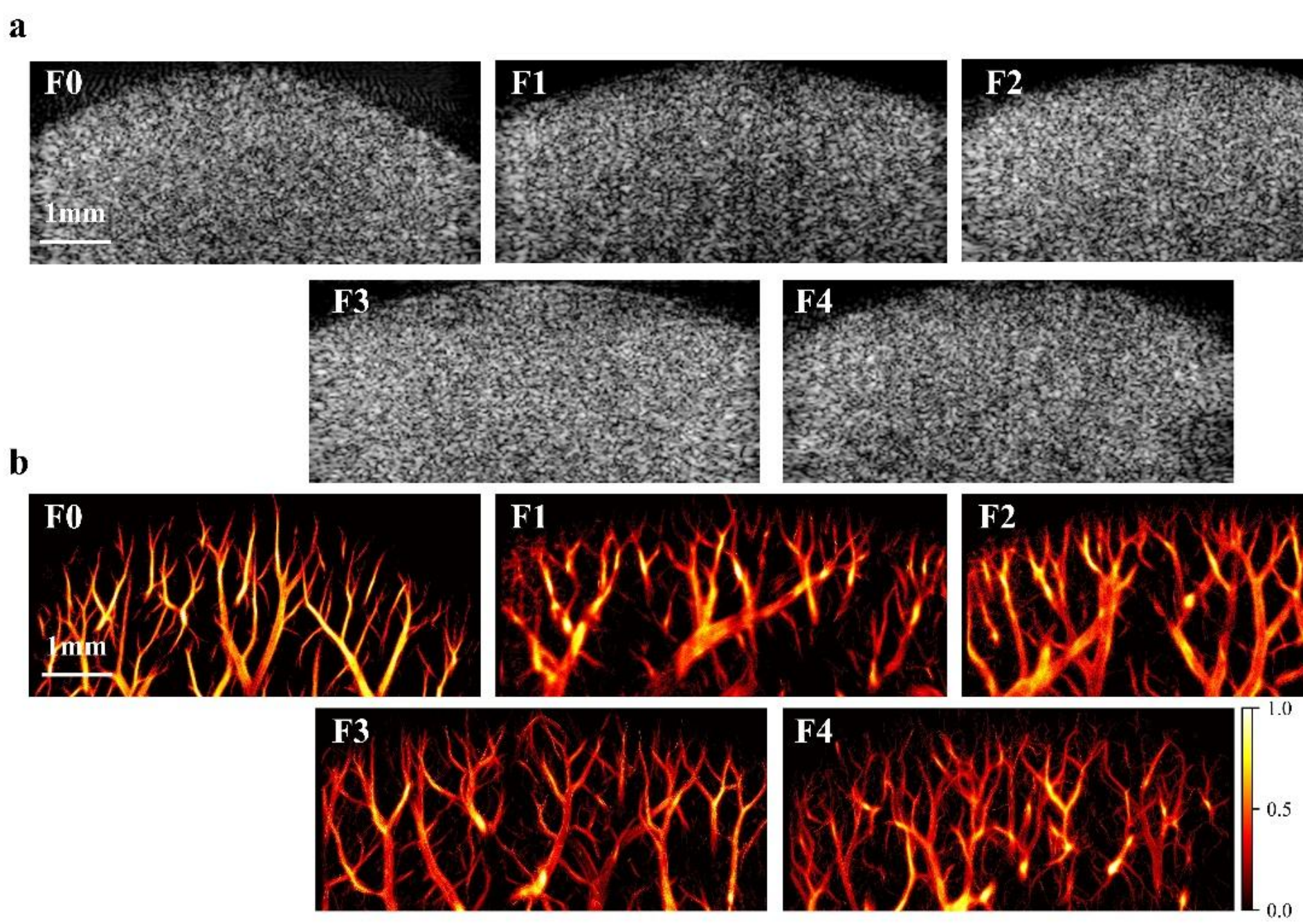

Supplement: Supplementary file 1 [file 41747_2026_841_MOESM1_ESM.pdf]
